# Loss of IL-33 enhances elastase-induced and cigarette smoke extract-induced emphysema in mice

**DOI:** 10.1186/s12931-021-01705-z

**Published:** 2021-05-15

**Authors:** Daisuke Morichika, Akihiko Taniguchi, Naohiro Oda, Utako Fujii, Satoru Senoo, Junko Itano, Arihiko Kanehiro, Yoshiaki Kitaguchi, Masanori Yasuo, Masayuki Hanaoka, Takashi Satoh, Shizuo Akira, Katsuyuki Kiura, Yoshinobu Maeda, Nobuaki Miyahara

**Affiliations:** 1 Independent Administrative Agency, National Hospital Organization, Fukuyama Medical Center, Fukuyama, Japan; 2 Department of Hematology, Oncology, Allergy and Respiratory Medicine, Okayama University Graduate School of Medicine, Dentistry, and Pharmaceutical Sciences, Okayama, Japan; 3 First Department of Internal Medicine, Shinshu University School of Medicine, Mastumoto, Japan; 4 Laboratory of Host Defense, World Premier Institute Immunology Frontier Research Center (WPI-IFReC), Osaka University, Osaka, Japan; 5 Department of Allergy and Respiratory Medicine, Okayama University Hospital, Okayama, Japan; 6 Department of Medical Technology, Okayama University Graduate School of Health Sciences, 2-5-1 Shikata-cho, Okayama, Okayama 700-8558 Japan

**Keywords:** Chronic obstructive pulmonary disease, COPD, HGF, VEGF

## Abstract

**Background:**

IL-33, which is known to induce type 2 immune responses via group 2 innate lymphoid cells, has been reported to contribute to neutrophilic airway inflammation in chronic obstructive pulmonary disease. However, its role in the pathogenesis of emphysema remains unclear.

**Methods:**

We determined the role of interleukin (IL)-33 in the development of emphysema using porcine pancreas elastase (PPE) and cigarette smoke extract (CSE) in mice. First, IL-33^−/−^ mice and wild-type (WT) mice were given PPE intratracheally. The numbers of inflammatory cells, and the levels of cytokines and chemokines in the bronchoalveolar lavage (BAL) fluid and lung homogenates, were analyzed; quantitative morphometry of lung sections was also performed. Second, mice received CSE by intratracheal instillation. Quantitative morphometry of lung sections was then performed again.

**Results:**

Intratracheal instillation of PPE induced emphysematous changes and increased IL-33 levels in the lungs. Compared to WT mice, IL-33^−/−^ mice showed significantly greater PPE-induced emphysematous changes. No differences were observed between IL-33^−/−^ and WT mice in the numbers of macrophages or neutrophils in BAL fluid. The levels of hepatocyte growth factor were lower in the BAL fluid of PPE-treated IL-33^−/−^ mice than WT mice. IL-33^−/−^ mice also showed significantly greater emphysematous changes in the lungs, compared to WT mice, following intratracheal instillation of CSE.

**Conclusion:**

These observations suggest that loss of IL-33 promotes the development of emphysema and may be potentially harmful to patients with COPD.

**Supplementary Information:**

The online version contains supplementary material available at 10.1186/s12931-021-01705-z.

## Background

Chronic obstructive pulmonary disease (COPD) is characterized by airflow obstruction that is not fully reversible, leading to respiratory insufficiency and functional disability [1, 2]. Existing therapeutic options for COPD can reduce the severity of symptoms, and the frequency and severity of exacerbations. However, these therapies have poor efficacy for maintaining lung function and palliating symptoms in COPD [1]. Therefore, new anti-inflammatory therapeutic strategies are required.

The pathogenesis of COPD remains unclear. It has been reported that macrophages, neutrophils, and T lymphocytes play important roles in the development and progression of emphysematous lung disease in humans. In contrast, human hepatocyte growth factor (HGF) has been reported to confer protection against the destruction of alveoli [3–5]; vascular endothelial growth factor (VEGF) signaling has also been reported to play a role in the maintenance of alveolar structures [6]. These observations suggest that determining the balance between destructive and protective factors in the lung is important to understand the pathogenesis of COPD and develop new therapeutic options for this disease.

Interleukin (IL)-33 is a member of the IL-1 family [7]. IL-33 not only induces type 2 immune response via group 2 innate lymphoid cells (ILC2s), stimulating basophils and eosinophils without an acquired immune response, but also induces proinflammatory cytokines, such as TNFα, IL-1β, and IL-6, via basophils and mast cells, and the Th1 cytokine IFN-γ via natural killer (NK) cells and NK T cells [7, 8].

The expression of IL-33 was reported to be increased in bronchial endothelial cells in a murine model of COPD [9]. IL-33 was shown to induce the expression of IL-6 and IL-8 following cigarette smoke extract (CSE) exposure [9]. Cigarette smoke (CS) has also been reported to induce bronchial epithelium-derived IL-33, and to decrease the expression of ST2 (the receptor of IL-33) on ILC2s and increase its expression on macrophages and NK cells [10]. Treatment with anti-IL-33 antibody has been reported to inhibit CS-induced airway inflammation [11]. These data suggest that IL-33 has important roles in the development of CS-induced airway inflammation, although its role in the development of emphysematous changes in the lung remains to be clarified.

The present study was performed to investigate the susceptibility of IL-33-deficient (IL-33^−/−^) mice to porcine pancreas elastase (PPE) and CSE, to clarify the role of IL-33 in the pathogenesis of emphysema.

## Methods

### Animals and experimental protocol

Female C57BL/6J mice were purchased from Charles River Japan, Inc. (Yokohama, Japan). IL33^−/−^ mice were generated as described by Yasuda et al. [12]. All animal experiments were performed in accordance with the guidelines of the Institutional Animal Care and Use Committee of Okayama University Medical School (Okayama, Japan).

### Elastase-induced emphysema model

To generate an elastase-induced emphysema model, female IL33^−/−^ mice (8–10 weeks old) and wild-type (WT) mice received 2.0 or 3.75 U of PPE by intratracheal instillation on day 0 [13]. Control mice received 40 µl of phosphate-buffered saline (PBS) at the same time point. The mice were sacrificed on days 0, 2, 4, 7, 14, and 21 to evaluate lung histology, lung function, airway inflammation, and the levels of cytokines and chemokines.

### Determination of static lung compliance

A flexiVent small-animal ventilator (SCIREQ, Montreal, QC, Canada) was used to assess static lung compliance (Cst), which is a measure of the elasticity of the lung. Cst was calculated from the pressure volume curves using flexiVent software (version 5.0; SCIREQ), as described previously [14]. Briefly, the mice were anesthetized by intraperitoneal injection of 150 mg/kg of ketamine and 10 mg/kg of xylazine (Kyoritsu Seiyaku, Tokyo, Japan). The mice were tracheostomized with a 5-mm section of metallic tubing (18 G cannula) and ventilated at 150 breaths/min with a tidal volume of 10 ml/kg and positive end-expiratory pressure of 3 cmH_2_O. The Cst was then measured.

### Bronchoalveolar lavage (BAL) fluid

Lungs were lavaged with Hanks’ balanced salt solution via the tracheal tube (2 × 1 ml, 37 °C), and the cells in BAL fluid were counted. Cytospin slides were stained and differentiated in a blinded manner by counting at least 200 cells under a light microscope, as described previously [15].

### Lung histology and morphometric measurements of airspace size

The left lung was inflated by intratracheal instillation of 10% formalin at a static pressure of 20 cmH_2_O and fixed in 10% formalin. The tissue was then embedded in paraffin and 2-µm-thick sections were stained with hematoxylin and eosin (H&E). The mean linear intercept (Lm), which represents the average size of alveoli, was calculated by counting lines of a defined length, as described previously [16–18].

### Measurement of cytokines and chemokines

The cytokine levels in the BAL fluid and supernatants of homogenized lungs were measured by enzyme-linked immunosorbent assay (ELISA), as described previously [19]. For preparation of lung homogenates, lung tissue was frozen at − 70 °C immediately after euthanasia. The lung tissue was mixed with 0.1% Triton-X100 solution in PBS containing proteinase inhibitors at a 1:2.5 ratio of weight per volume (Sigma-Aldrich, St. Louis, MO, USA). The specimens were homogenized and then centrifuged at 21,480×*g* for 30 min. The supernatants were frozen at − 80 °C until analysis. The limits of detection were 2 pg/ml for keratinocyte-derived chemokine (KC), 2.0 pg/ml for monocyte chemoattractant protein-1 (MCP-1), 1.5 pg/ml for macrophage inflammatory protein-2 (MIP-2), 12.1 pg/ml for HGF, 3.0 pg/ml for VEGF, and 0.014 ng/ml for matrix metalloproteinase-9 (MMP-9) (R&D Systems, Minneapolis, MN, USA).

### Lung cell isolation

Lungs of PPE-treated mice were placed in PBS containing heat-inactivated 10% FCS. Lung tissue was minced and incubated for 1 h at 37 °C in 5 ml of PBS containing 0.05% collagenase I (Sigma-Aldrich), and then dispersed by passing through a 20 G needle several. The suspensions were strained through a cell strainer. The pulmonary mononuclear cells were isolated by density gradient cell centrifugation over Histopaque (Sigma-Aldrich) [20].

### Flow cytometry

#### Analyses of ILC2s

The cells isolated from digested lungs were stained with biotin-conjugated antibody mixtures for lineage markers (CD4, CD5, CD8, CD11c, CD11b, CD19, NK1.1, Gr-1, TER119, FcεRI, and B220), Pacific blue-conjugated anti-Sca-1, PECy7-conjugated c-Kit (CD117), APC-conjugated anti-IL-7Rα (CD127), FITC conjugated anti-T1/ST2, APC-Cy7-conjugated anti-CD25, and PE conjugated anti-streptavidin, and analyzed using a MACSQuant Analyzer (Miltenyi Biotec, Bergisch Gladbach, Germany). Lin^−^Sca^+^c-Kit^+^IL-7R^+^CD25^+^ST2^dim^ cells were identified as lung ILC2s [21, 22]. The data were analyzed using FlowJo (TreeStar, Ashland, OR, USA). APC-Cy7-conjugated anti-CD25, Pacific blue-conjugated anti-Sca-1, biotin-conjugated anti-CD4, anti-CD5, anti-CD8, anti-CD11b, anti-NK1.1, anti-Gr-1, anti-TER119, and anti-B220, and PE-conjugated anti-streptavidin antibodies were obtained from BD Biosciences (Franklin Lakes, NJ, USA). FITC-conjugated anti-T1/ST2 was from MD Bioscience (St Paul, MN, USA). APC-conjugated anti-IL-7Rα and biotin-conjugated anti-FcεRI antibodies were from BioLegend (San Diego, CA, USA). PECy7-conjugated c-Kit was from eBioscience (La Jolla, CA, USA). Biotin-conjugated anti-CD11c and anti-CD19 were from TONBO Biosciences (San Diego, CA, USA).

### IL-33 receptor ST2 blockade with anti-ST2 antibody

WT mice received PPE or PBS via intratracheal instillation on day 0. The mice received an intraperitoneal injection of 100 µg anti-ST2 antibody (R&D Systems) on days 1, 3, 6, and 9. Control mice received 100 µg rat-IgG (R&D Systems) at the same time points. Lung function and lung histology were evaluated on day 21.

### Administration of recombinant IL-33 (rIL-33)

WT mice received PPE or PBS by intratracheal instillation on day 0. The mice received an intraperitoneal injection of 1000 ng of rIL-33 (R&D Systems) on days 0 and 3. Control mice received PBS at the same time points. Lung function and lung histology were evaluated on day 21. In some experiments, IL33^−/−^ mice received an intraperitoneal injection of 1000 ng rIL-33 on day 0 following intratracheal instillation of PPE or PBS. Control mice received PBS at the same time point. BAL was performed on day 2, and the levels of HGF and VEGF in BAL fluid were measured.

### CSE-induced emphysema model

CSE was prepared using the 3R4F Kentucky reference cigarette. One non-filtered cigarette was burned; the smoke was passed through 8 ml of PBS using a vacuum pump with a constant air flow of 2 l/min. The extract was used when pH was between 6.2 and 6.4 and the optical density at 320 nm (OD_320_) was between 1.600 and 1.900. The extract was freshly prepared for each experiment and administered after the pH had been adjusted between 7.00 and 7.40 and the solution had been filtered through a 0.2-μm pore-size filter to remove particles and bacteria [14].

The experimental mice received 40 µl of CSE intratracheally on days 0, 7, and 14. Control mice received 40 µl of PBS at the same time points. The mice were sacrificed on day 21 to evaluate lung histology. In some experiments, the mice received 400 µl of CSE intraperitoneally on days 0, 7, 14, and 21. The control mice received 400 µl of PBS at the same points. The mice were sacrificed on day 28 to evaluate lung histology [14].

### Statistical analysis

All results are presented as the mean ± standard error of the mean (SEM). Groups were compared by one-way analysis of variance (ANOVA). Pairs of samples with a parametric distribution were compared by unpaired two-tailed Student’s *t* test, and samples with a nonparametric distribution were compared by the Mann–Whitney U test. In all analyses, *P* < 0.05 was taken to indicate statistical significance.

## Results

### IL-33 levels in the lung are increased following PPE instillation

**Fig. 1:**
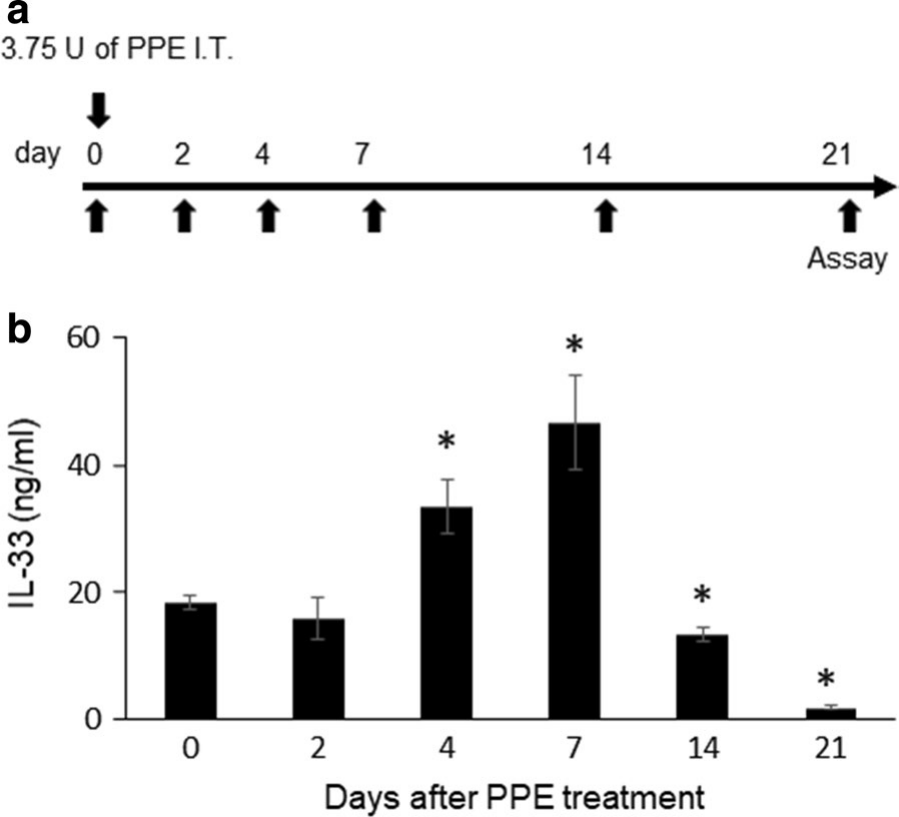
Kinetics of IL-33 in the lungs of porcine pancreas elastase (PPE)-treated WT mice. **a** Experimental protocol. **b** Kinetics of IL-33 in the lung. The results for each group are shown as the mean ± SEM; *n* = 7–9 in each group. *P < 0.05 compared to naïve mice. Day 0 indicates the group without PPE instillation

We first examined whether administration of PPE affected the levels of IL-33 in the lung. We measured the kinetics of IL-33 in the lung homogenate of PPE-instilled WT mice by ELISA (Fig. 1a). The levels of IL-33 in the lung were significantly increased on days 4 and 7 following instillation of PPE compared to those on day 0 (Fig. 1b).

### Development of elastase-induced emphysema is enhanced in IL-33^−/−^ mice

**Fig. 2:**
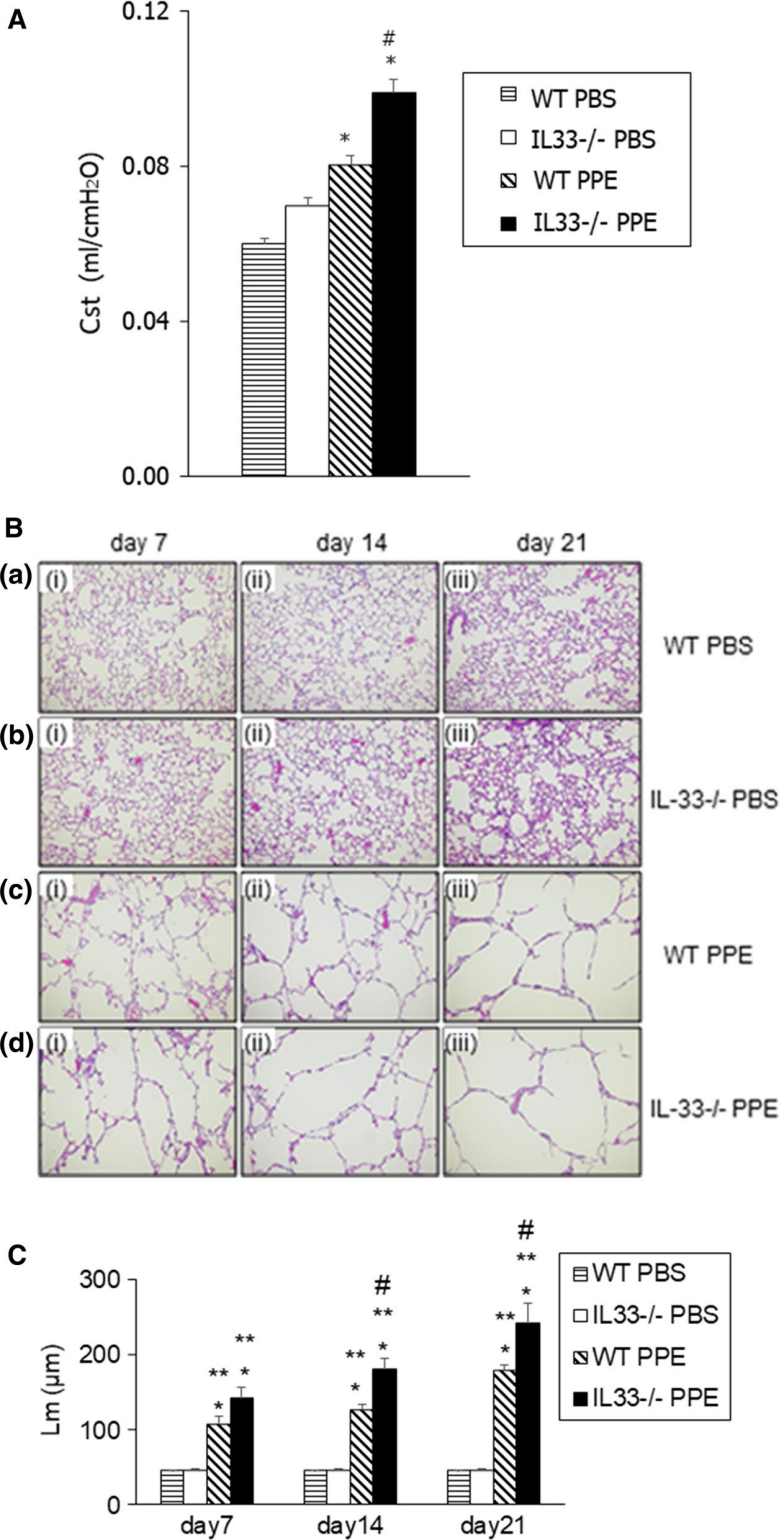
Assessment of PPE-induced emphysema in IL-33^−/−^ mice. **a** Static lung compliance (Cst) values in WT and IL-33^−/−^ mice on day 21 after intratracheal instillation of PPE or saline. Mice received 2 U of PPE or saline by intratracheal instillation. Cst values were obtained as described in Materials and Methods. WT PBS: WT mice treated with PBS. IL-33^−/−^ PBS: IL-33^−/−^ mice treated with PBS. WT PPE: WT mice treated with PPE. IL-33^−/−^ PPE: IL-33^−/−^ mice treated with PPE. Data are shown as the mean ± SEM (n = 5–10 in each group). *P < 0.05 compared to WT PBS; #P < 0.05 compared to WT PPE. **b** Representative images of H&E-stained lung tissue (magnification: × 200) (a) WT mice treated with PBS on (i) day 7, (ii) day 14, and (iii) day 21, (b) IL-33^−/−^ mice treated with PBS on (i) day 7, (ii) day 14, and (iii) day 21, (c) WT mice treated with PPE on (i) day 7, (ii) day 14, and (iii) day 21, (d) IL-33^−/−^ mice treated with PPE on (i) day 7, (ii) day 14, and (iii) day 21. **c** Mean linear intercept (Lm) alveoli values. Morphometric assessment was performed on days 7, 14, and 21 after instillation. Lm values were determined as described in the “Materials and methods”. Data are shown as the mean ± SEM (*n* = 5–10 in each group). *P < 0.05 compared to WT PBS; **P < 0.05 compared to IL-33^−/−^ PBS; ^#^*P* < 0.05 compared to WT PPE

We assessed lung function to clarify the role of IL-33 in the development of elastase-induced emphysema. Cst values were higher in PPE-instilled WT mice than in saline-instilled WT or IL-33^−/−^ mice on day 21. The Cst value was significantly higher in PPE-instilled IL-33^−/−^ mice than PPE-instilled WT mice (Fig. 2a). Lung histology indicated airspace enlargement and alveolar wall destruction in PPE-instilled WT mice (Fig. 2b). These emphysematous changes were markedly increased in PPE-instilled IL-33^−/−^ mice (Fig. 2b). To further assess the development of emphysema, we monitored airspace enlargement by determining the Lm in H&E-stained tissue sections. Lm values in PPE-instilled WT mice were significantly increased compared to saline-instilled WT mice. These changes in PPE-instilled IL-33^−/−^ mice were significantly enhanced compared to those in PPE-instilled WT mice on days 7 and 14 (Fig. 2c). The results of these histological analyses suggested that IL-33^−/−^ mice have increased susceptibility to elastase-induced emphysema compared to WT controls.

### Elastase-induced airway inflammation in WT and IL-33^−/−^ mice

**Fig. 3:**
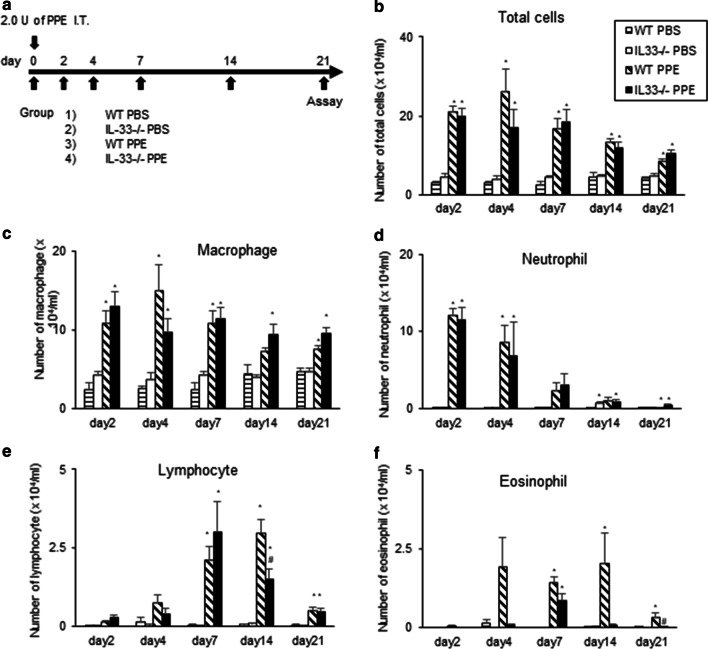
Cell composition in BAL fluid on days 2, 7, 14, and 21 after PPE treatment. **a** Experimental protocol. Numbers of **b** total cells, **c** macrophages, **d** neutrophils, **e** lymphocytes, and **f** eosinophils. The results for each group are shows as the mean ± SEM; *n* = 5–10 in each group. *P < 0.05 compared to WT PBS; ^#^*P* < 0.05 compared to WT PPE

Airway inflammation is thought to be a major contributor to the development of emphysema [20]. We assessed the severity of the inflammatory response by measuring cell accumulation in BAL fluid at different time points (Fig. 3a). In WT mice, the recruitment of inflammatory cells into airways was increased following instillation of PPE (Fig. 3b). The total number of cells in BAL fluid was increased largely due to increased numbers of neutrophils and macrophages from day 2 to day 21 (Fig. 3c and d). There was no significant difference in the number of these cells between PPE-instilled WT mice and PPE-instilled IL-33^−/−^ mice.

### Cytokine and chemokine levels in BAL fluid

**Fig. 4:**
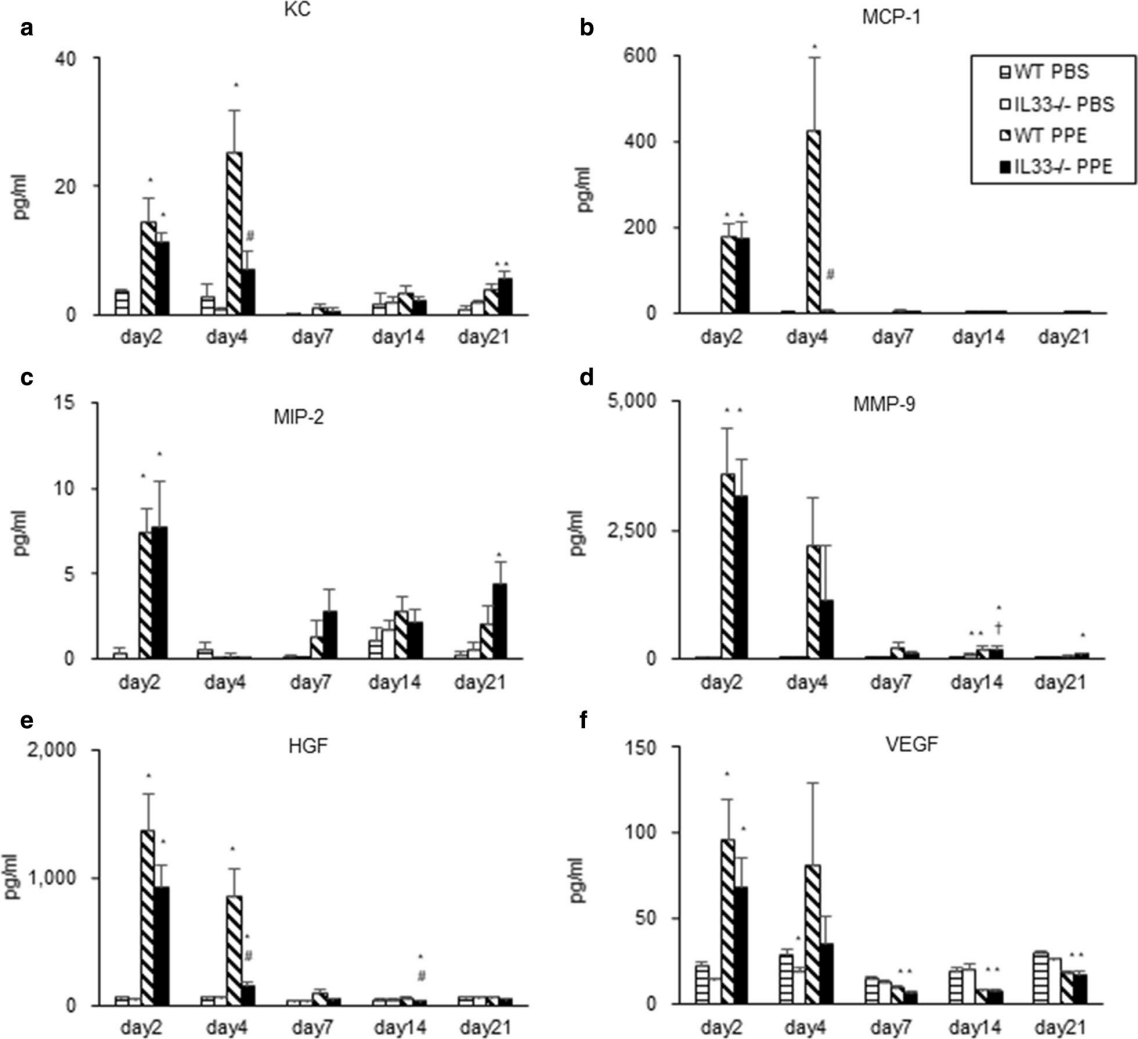
Cytokine and chemokine levels in BAL fluid on days 2, 7, 14, and 21 after PPE treatment. **a** KC, **b** MCP-1, **c** MIP-2, **d** MM-9, **e** HGF, and **f** VEFG. The results for each group are shows as the mean ± SEM; *n* = 4–6 in each group. *P < 0.05 compared to WT PBS. ^†^*P* < 0.05 compared to IL33^−/−^ PBS; ^#^*P* < 0.05 compared to WT PPE

To further determine the mechanisms underlying the increased susceptibility of PPE-instilled IL-33^−/−^ mice to PPE-induced emphysema, we measured the levels of neutrophil- and macrophage-related chemokines and proteinases related to the destruction of alveoli [13, 20, 23], as well as HGF and VEGF, which protect against the development of emphysema [5, 6], in BAL fluid at different time points. The levels of KC and MCP-1 were significantly increased in PPE-instilled WT mice compared to PBS-instilled mice on days 2 and 4 (Fig. 4a and b). The levels were also increased in PPE-instilled IL-33^−/−^ mice on day 2, but were significantly lower than in PPE-instilled WT mice on day 4. The levels of MIP-2 and MMP-9 were not different between IL-33^−/−^ mice and WT mice following PPE instillation (Fig. 4c and d). The levels of HGF and VEGF were significantly increased in PPE-instilled WT mice compared to those in PBS-instilled mice on days 2 and 4 (Fig. 4e and f). The levels of HGF in PPE-instilled IL-33^−/−^ mice were significantly reduced compared to PPE-instilled WT mice on day 4. The levels of VEGF in PPE-instilled IL-33^−/−^ tended to be lower on day 4, but the difference between the two strains of mice was not significant.

### ILC2s in lung tissue

**Fig. 5:**
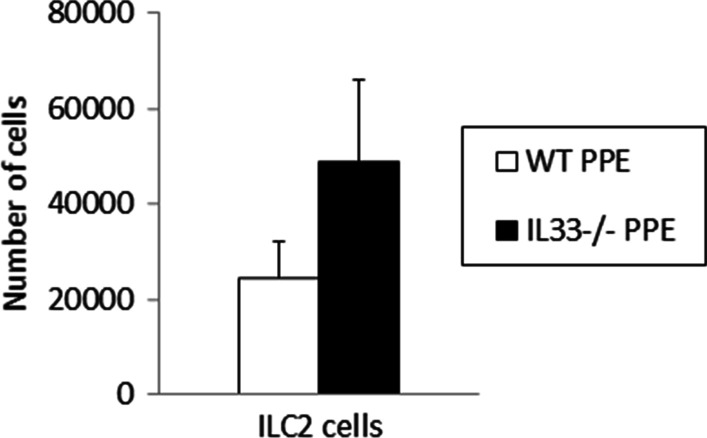
Number of ILC2s in the lung on day 7 after PPE treatment. The results for each group are shown as the mean ± SEM; *n* = 7 in each group. There was no significant difference in the number of ILC2s in the lung between PPE-treated WT mice and PPE-treated IL33^−/−^mice

IL-33 is a cytokine that recruits ILC2s into the airway. We examined whether ILC2s play an important role in PPE-induced emphysema. As show*n* in Fig. 2b, the levels of IL-33 in lung homogenates were increased following PPE instillation on day 7 in our murine model. We counted the ILC2s in the lung on day 7. The number of ILC2s tended to be higher in PPE-instilled IL-33^−/−^ mice compared to PPE-instilled WT mice, but the difference was not significant (Fig. 5).

### Treatment with anti-ST2 antibody in PPE-instilled mice

We assessed whether blockade of the IL-33 receptor, ST2, affected PPE-induced emphysema in WT mice. Administration of anti-ST2 antibody did not affect the Cst values or the MLI values in PPE-instilled mice (Additional file 1:  Fig. S1). Although complete loss of IL-33 may enhance the development of emphysema, partial blockade of IL-33 does not appear to promote emphysematous changes in the lung.

### Treatment with rIL-33 does not reduce emphysema in PPE-instilled mice

**Fig. 6:**
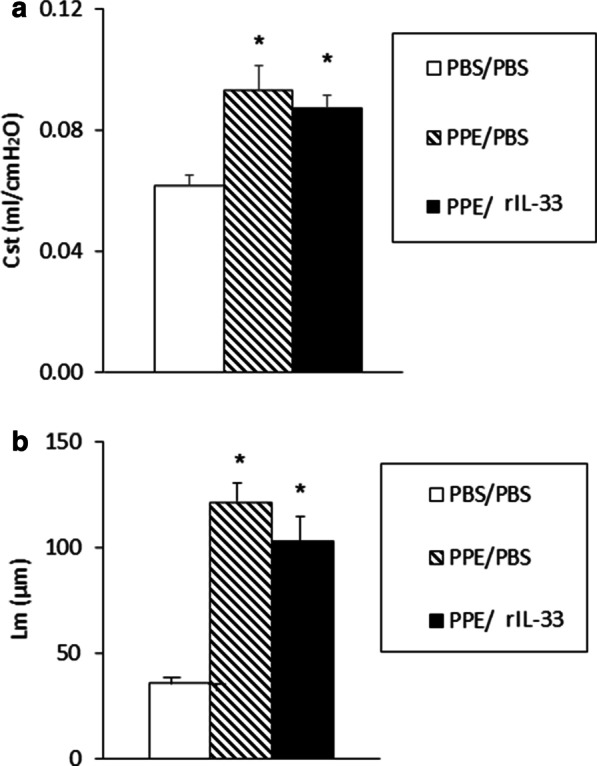
Treatment of PPE-induced emphysema with recombinant IL-33. The mice received intraperitoneal injection of recombinant IL-33 (rIL-33) or PBS on days 0 and 3 following instillation of PBS or PPE. Lung function, lung histology, and morphometric measurements were performed 21 days as described in the Materials and Methods. **a** Cst values following treatment with rIL-33. **b** Lm values. Data are shown as mean ± SEM (n = 8 in each group). PBS/PBS: PBS-instilled mice treated with PBS. PPE/PBS: PPE-instilled mice treated with PBS. PPE/rIL-33: PPE-instilled mice treated with rIL-33. *P < 0.05 compared to PBS/PBS. There were no significant differences between PPE/PBS and PPE/rIL-33

As loss of IL-33 enhances emphysematous changes, we examined whether IL-33 had a protective effect on PPE-induced emphysema in WT mice. The Cst values did not decrease following administration of rIL-33 to mice that received PPE (Fig. 6a). Emphysematous changes in lung pathology were unaffected (Fig. 6b). These data suggest that although complete loss of IL-33 enhances the development of emphysema, IL-33 itself may not protect against the development of emphysematous changes in the lung.

### rIL-33 treatment increased HGF levels in the airway of PPE-instilled IL-33^−/−^ mice

**Fig. 7:**
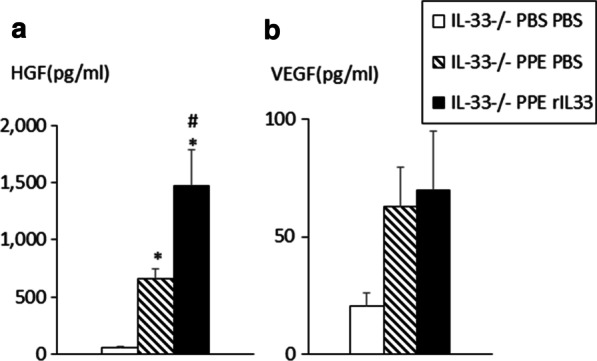
HGF and VEGF levels in BAL fluid of IL-33^−/−^ mice following treatment with rIL-33. The IL-33^−/−^ mice received intraperitoneal injection of rIL-33 or PBS on day 0 following instillation of PBS or PPE. BAL were performed on day 2, and levels of HGF and VEGF in BAL fluid were measured as described in the Materials and Methods. **a** HGF levels. **b** VEGF levels. Data are shown as the mean ± SEM (*n* = 5–8 in each group). IL-33^−/−^ PBS PBS: PBS-instilled IL-33^−/−^ mice treated with PBS. IL-33^−/−^ PPE PBS: PPE-instilled IL-33^−/−^ mice treated with PBS. IL-33^−/−^ PPE rIL-33: PPE-instilled IL-33^−/−^ mice treated with rIL-33. *P < 0.05 compared to IL-33^−/−^ PBS PBS. ^#^P < 0.05 compared to IL-33^−/−^ PPE PBS

The HGF levels in PPE-instilled IL-33^−/−^ mice were significantly lower than PPE-instilled WT mice. Thus, we examined whether rIL-33 treatment increased HGF levels in IL-33^−/−^ mice. The mice treated with rIL-33 had higher HGF levels in BAL fluid than mice treated with PBS following PPE instillation (Fig. 7a). The VEGF levels in BAL fluid of rIL-33-treated mice were not significantly different from those of PBS-treated mice (Fig. 7b). These data suggest that IL-33 induced HGF secretion in the airway.

### CSE-induced emphysema is enhanced in IL-33^−/−^ mice

Animal models of emphysema induced by CS or CSE more accurately represent emphysematous disease in humans than those in which emphysema is induced by PPE. We further assessed whether the emphysematous changes in IL-33^−/−^ mice were enhanced in the CSE-induced emphysema model. We have recently reported that intraperitoneal injection of CSE induces emphysematous changes in rat [17] and mice [14]. Following intraperitoneal injection of CSE, IL-33^−/−^ mice showed significantly enhanced emphysematous changes compared WT mice (Additional file 2:  Fig. S2a–c).

**Fig. 8:**
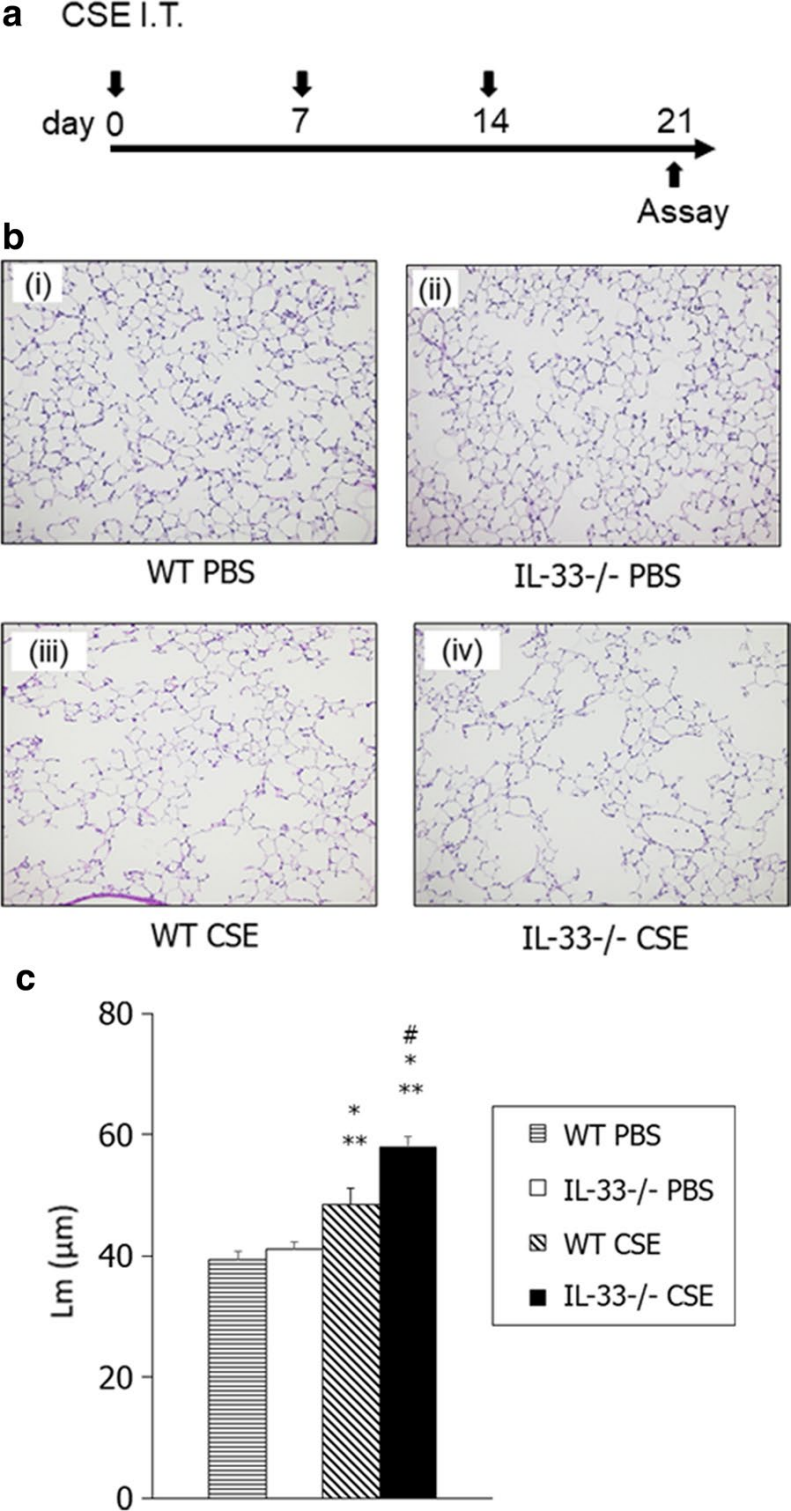
Assessment of cigarette smoke extract (CSE)-induced emphysema. **a** Experimental protocol. **b** Representative images of H&E-stained lung tissue (magnification: × 200). **c** Lm values. WT PBS: WT mice treated with PBS. IL-33^−/−^ PBS: IL-33^−/−^ mice treated with PBS. WT CSE: WT mice treated with CSE. IL-33^−/−^ CSE: IL-33^−/−^ mice treated with CSE. Results for each group are shown as mean ± SEM (n = 7–8 in each group). *P < 0.05 compared to WT PBS; **P < 0.05 compared to IL-33^−/−^ PBS; ^#^*P* < 0.05 compared to WT CSE

We next assessed the ability of intratracheal CSE to induce emphysematous changes in the lung (Fig. 8a). Airspace enlargement and alveolar wall destruction were observed in intratracheally CSE-treated WT mice in lung histology. These emphysematous changes were significantly increased in CSE-treated IL-33^−/−^ mice compared to CSE-treated WT mice (Fig. 8b). Lm values in CSE-treated WT mice were significantly increased compared to saline-treated WT mice. These changes in CSE-treated IL-33^−/−^ mice were significantly enhanced compared to those in CSE-treated WT mice on day 21 (Fig. 8c). These histological observations indicated that IL-33^−/−^ mice are more susceptible to CSE-induced emphysema than WT mice.

## Discussion

IL-33 is a member of the IL-1 family, which acts as a ligand for ST2 (IL-1Ra) [7] and induces not only type 2 cytokines, but also proinflammatory cytokines, such as IL-1β, IL-6, and TNF-α, from mast cells [24–27] and basophils [8], as well as the type-1 cytokine IFN-γ from NK cells and NKT cells [28, 29]. These proinflammatory cytokines, together with Th1-related immunity, play important roles in the pathogenesis of COPD [30]. Treatment with anti-IL-33 antibody has been reported to inhibit CS-induced airway inflammation [11]. These studies suggested that IL-33 is involved in the pathogenesis of COPD, especially airway inflammation. However, the role of IL-33 in the development of emphysematous changes in the lung remains to be elucidated.

In the present study, to clarify the role of IL-33 in the development of emphysematous changes in the lung, we investigated both PPE- and CSE-induced emphysematous changes in the lungs of IL-33^−/−^ and WT mice, as well as the kinetics of cytokines and chemokines in the lung. Our results demonstrated that airspace enlargement of the lung was significantly enhanced in IL-33^−/−^ mice compared to WT mice, in both PPE and CSE-induced emphysema models. There was no significant difference between PPE-treated IL-33^−/−^ and WT mice in the number of macrophages or neutrophils in BAL fluid. Instead, the level of HGF, which protects against the development of emphysema, was significantly lower in IL-33^−/−^ mice compared to WT mice following PPE instillation. These observations suggested that loss of IL-33 may enhance the development of elastase-induced and CSE-induced emphysematous changes.

IL-33 is a cytokine that induces a Th2 cell immune response via ILC2s [7]. On the other hand, in the pathogenesis of COPD, IL-33 may mainly affect macrophages and NK cells, because it has been reported that CS reduces the expression of ST2 on ILC2s and increases its expression on macrophages and NK cells [10]. In the present study, the levels of IL-33 in the lung were significantly increased following PPE instillation, but the number of ILC2s in the lung was not increased by PPE treatment when the level of IL-33 in the lung reached its peak. These observations suggested that IL-33 did not affect ILC2s in our model.

The accumulation of inflammatory cells, including neutrophils, macrophages, and CD8^+^ T cells, along with proteinase/anti-proteinase imbalance, apoptosis, and oxidative stress, may play an important role in the pathogenesis of COPD [30]. Neutrophils, which secrete MMP-9, cathepsin G, and neutrophil elastase, are predictors of disease severity with respect to the emphysematous component of COPD [31]. Human MMP-9 was shown to induce emphysema in a murine model [32], and MMP-9/TIMP-1 imbalance was observed in patients with COPD [33]. These observations suggest that controlling neutrophilic inflammation and MMPs may prevent the progression of emphysema. IL-33 expression is induced by exposure to CS in bronchial endothelial cells, and may in turn induce the expression of IL-6 and IL-8 [9]. Treatment with anti-IL-33 antibody has been reported to inhibit CS-induced airway inflammation [11]. These observations suggest that IL-33 may play an important role in the development of CS-induced airway inflammation. In the present study, however, IL-33 deficiency did not affect PPE-induced neutrophilic airway inflammation. The levels of neutrophilic chemoattractants, such as KC and MCP, were lower in IL-33^−/−^ mice compared to WT mice following PPE instillation. These observations suggest that although partial loss of IL-33 could attenuate airway inflammation, as reported previously [10, 11], complete loss of IL-33 does not improve the proteinase/anti-proteinase imbalance.

To further elucidate the mechanisms by which deficiency of IL-33 exacerbates emphysematous changes in the lung, we assessed lung-protective factors. It has been reported that VEGF and HGF protect against the development of emphysema. HGF recruited stem cells into injured tissue and induced their differentiation into tissue-specific cells [34]. Treatment with HGF reversed lung emphysema, possibly through stem cell mobilization and alveolar regeneration [3, 4]. Our data showed that the level of HGF in BAL fluid was significantly increased in PPE-treated WT and IL33^−/−^ mice compared to PBS-treated mice, and the increase in HGF level in PPE-treated IL33^−/−^ mice was less persistent compared to that in PPE-treated WT mice. In addition, administration of rIL-33 increased the levels of HGF in BAL fluid of PPE-instilled mice. These data suggest that IL-33 may protect against the development of elastase-induced emphysema via HGF expression. It has also been reported that loss of VEGF amplified the IL-33-mediated inflammatory response [35]. Inhibition of VEGF decreased alveolarization and induced alveolar septal cell apoptosis, but did not inhibit lung cell proliferation [6, 36, 37]. In the present study, however, VEGF levels in the lungs were not different between IL-33^−/−^ and WT mice following instillation of PPE or CS; therefore, VEGF may not have been affected in our emphysema models.

This study had some limitations. First, although IL-33-/- mice displayed enhanced emphysematous changes, this only occurred when IL-33 was completely deficient. Treatment with anti-ST2 receptor antibody did not enhance emphysema development, suggesting that partial IL-33 blockade is not sufficient to induce emphysema. Whether blockade of the IL-33-ST2 axis is harmful or beneficial for COPD in the clinical setting remains unclear. Second, treatment with IL-33 did not significantly suppress emphysema development. However, modifications to the dose and timing of IL-33 administration may produce different outcomes. Thus, the effects of IL-33 supplementation for COPD remain unclear. Third, we have not assessed the CS-induced emphysema model, which more closely mimics human emphysema. We have shown that a congenital IL-33 defect might enhance elastase- and CSE-induced emphysema. Collectively, our results indicate that clinical manipulations of the IL-33-ST2 axis may have varying effects on lung structure.

## Conclusions

We demonstrated that the loss of IL-33 may enhance the development of emphysema. Our data suggested that although IL-33 may affect CS-induced airway inflammation, complete loss of IL-33 may enhance emphysema and potentially be harmful in patients with COPD.

## Supplementary Information

**Additional file 1.** Treatment with anti-ST2 antibody in PPE-instilled mice. The mice received intraperitoneal injection of anti-ST2 antibody or IgG antibody on days -1, 1, 2, 4, 7, and 10 following instillation of PBS or PPE. BAL and lung morphometric measurements were performed on day 21 as described in the Materials and Methods. (A) Cell composition in BAL fluid. (B) Lm values. Data are shown as the mean ± SEM (n = 8 in each group). PBS/IgG: PBS-instilled mice treated with IgG. PPE/IgG: PPE-instilled mice treated with IgG. PPE/anti-ST2: PPE-instilled mice treated with anti-ST2 antibody. *P < 0.05 compared to PBS/PBS; There were no significant differences between PPE/IgG and PPE/anti-ST2.

**Additional file 2. ** Morphometric assessment of emphysema induced by intraperitoneal administration of cigarette smoke extract (CSE). The experimental protocol.

**Additional file 3.** (B) Representative images of H&E-stained lung tissue (magnification: ×200).

**Additional file 4.** (C) Lm values. WT PBS: WT mice treated with PBS. IL-33-/- PBS: IL-33-/- mice treated with PBS. WT CSE: WT mice treated with CSE. IL-33-/- CSE: IL-33-/- mice treated with CSE. The results for each group are shown as the mean ± SEM; n = 6–9 in each group. *P < 0.05 compared to WT PBS; **P < 0.05 compared to IL-33−/− PBS; #P < 0.05 compared to WT PPE.

## Abbreviations

ANOVA: One-way analysis of variance
BAL: Bronchoalveolar lavage
COPD: Chronic obstructive pulmonary disease
CS: Cigarette smoke
CSE: Cigarette smoke extract
Cst: Static lung compliance
CXCL5: C-X-C motif ligand 5
ELISA: Enzyme-linked immunosorbent assay
H&E: Hematoxylin and eosin
HGF: Hepatocyte growth factor
IL: Interleukin
ILC2: Group 2 innate lymphoid cell
KC: Keratinocyte chemoattractant
Lm: Mean linear intercept
MCP-1: Chemoattractant protein-1
MIP-2: Macrophage inflammatory protein-2
NK: Natural killer
MMP-9: Matrix metalloproteinase-9
PBS: Phosphate-buffered saline
PPE: Porcine pancreas elastase
TIMP-1: Tissue inhibitor of metalloproteinase-1
TNF-α: Tumor necrosis factor-alpha
VEGF: Vascular endothelial growth factor
WT: Wild-type
