# Supplementary figures and images for: Loss of IL-33 enhances elastase-induced and cigarette smoke extract-induced emphysema in mice
Source: Respir Res. 2021 May 15;22:150. doi: 10.1186/s12931-021-01705-z (PMC8122555; doi:10.1186/s12931-021-01705-z)

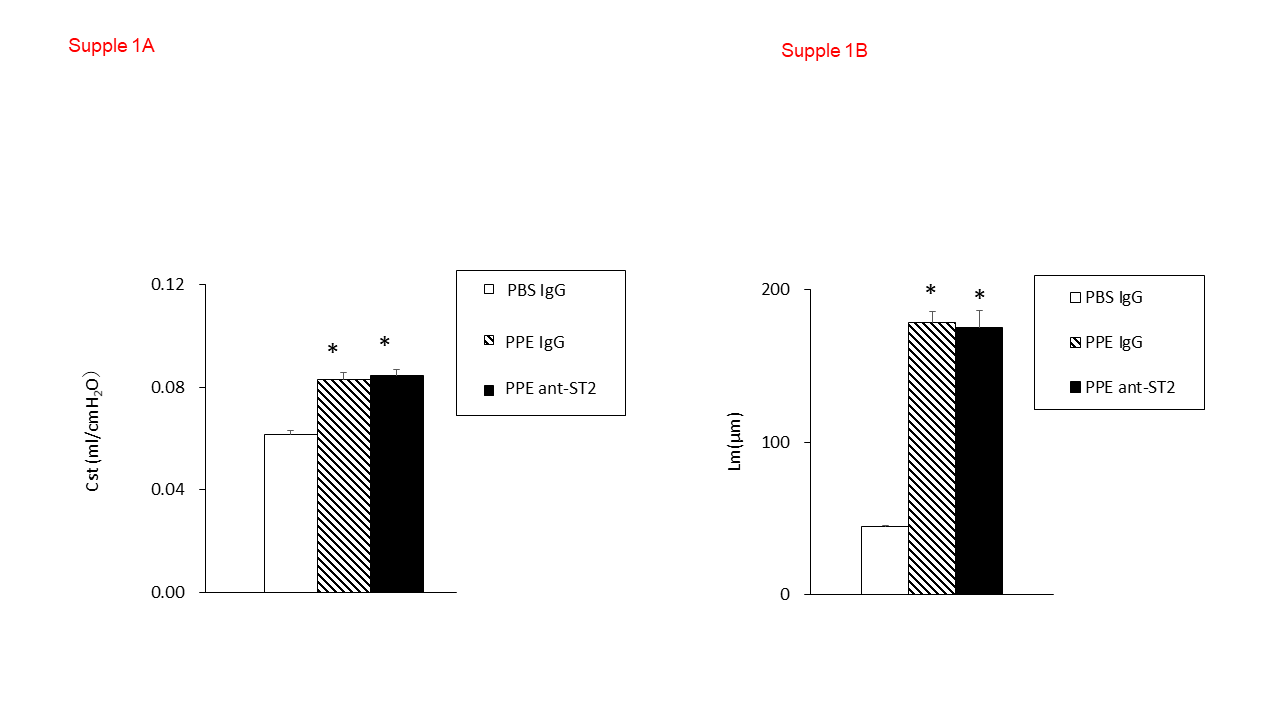

Supplement: Supplementary file 1 — Additional file 1. Treatment with anti-ST2 antibody in PPE-instilled mice. The mice received intraperitoneal injection of anti-ST2 antibody or IgG antibody on days -1, 1, 2, 4, 7, and 10 following instillation of PBS or PPE. BAL and lung morphometric measurements were performed on day 21 as described in the Materials and Methods. (A) Cell composition in BAL fluid. (B) Lm values. Data are shown as the mean ± SEM (n = 8 in each group). PBS/IgG: PBS-instilled mice treated with IgG. PPE/IgG: PPE-instilled mice treated with IgG. PPE/anti-ST2: PPE-instilled mice treated with anti-ST2 antibody. *P < 0.05 compared to PBS/PBS; There were no significant differences between PPE/IgG and PPE/anti-ST2. [file 12931_2021_1705_MOESM1_ESM.tif]

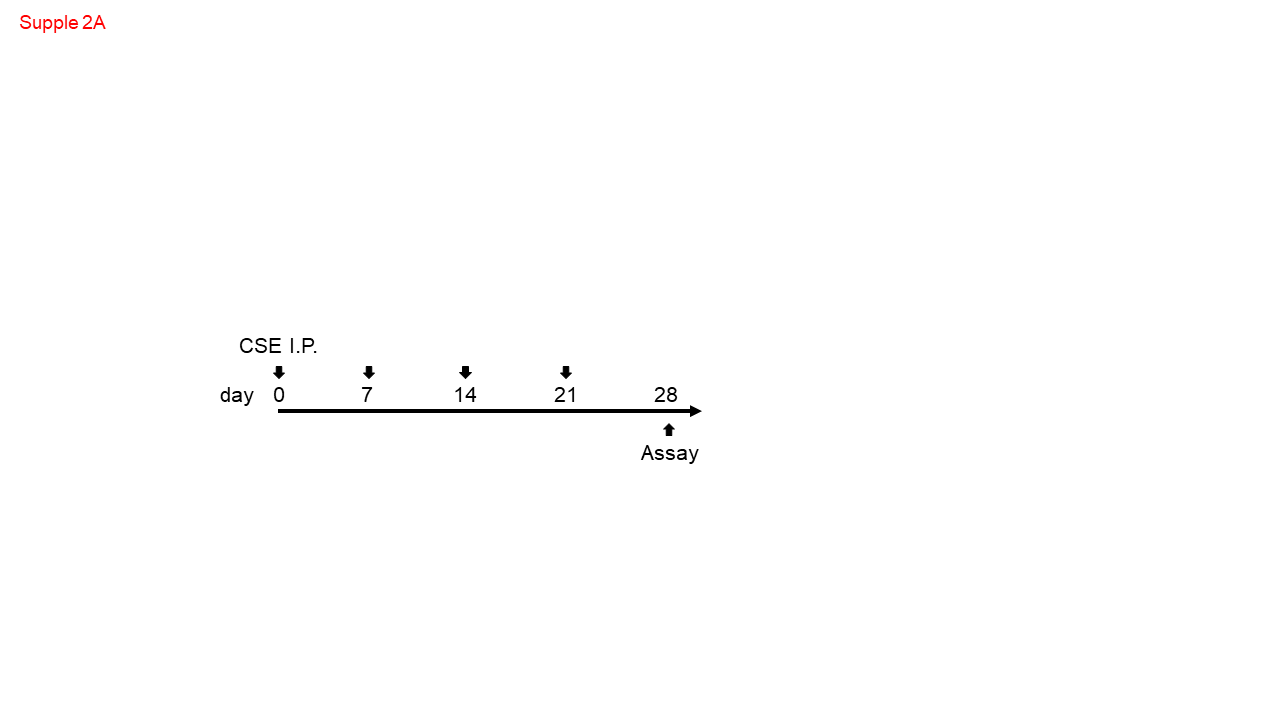

Supplement: Supplementary file 2 — Additional file 2. Morphometric assessment of emphysema induced by intraperitoneal administration of cigarette smoke extract (CSE). The experimental protocol. [file 12931_2021_1705_MOESM2_ESM.tif]

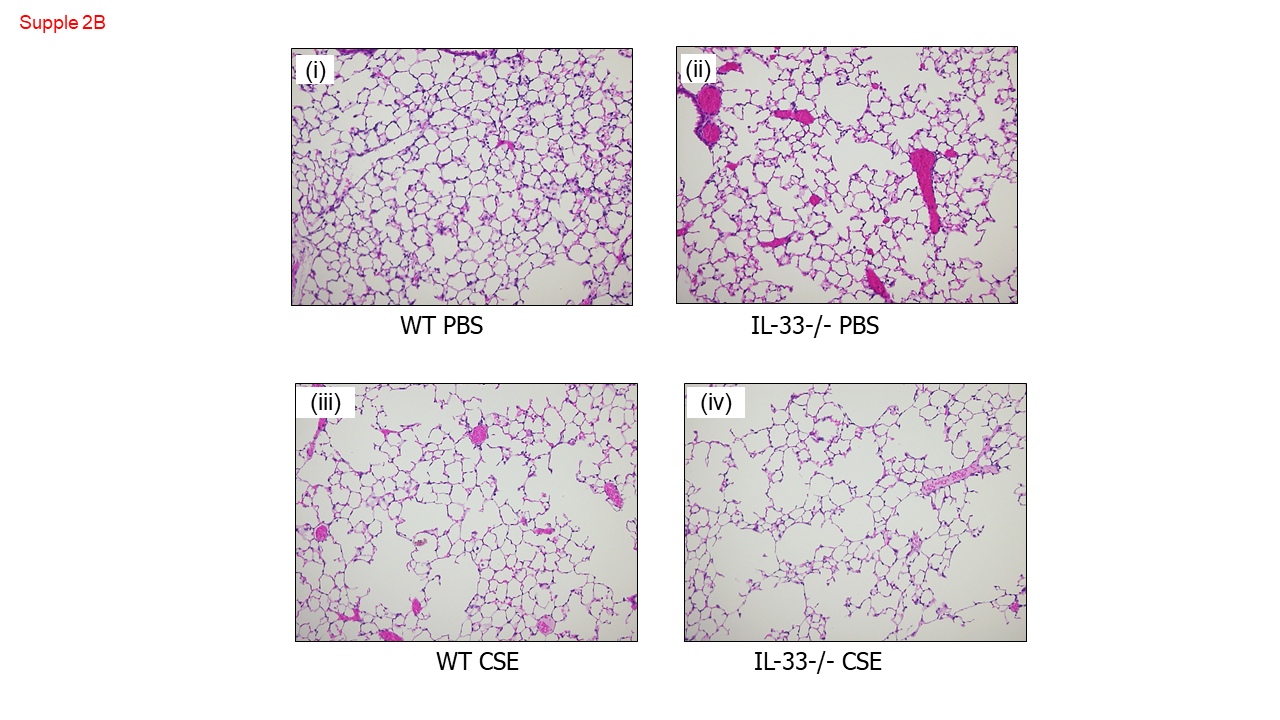

Supplement: Supplementary file 3 — Additional file 3. (B) Representative images of H&E-stained lung tissue (magnification: ×200). [file 12931_2021_1705_MOESM3_ESM.tif]

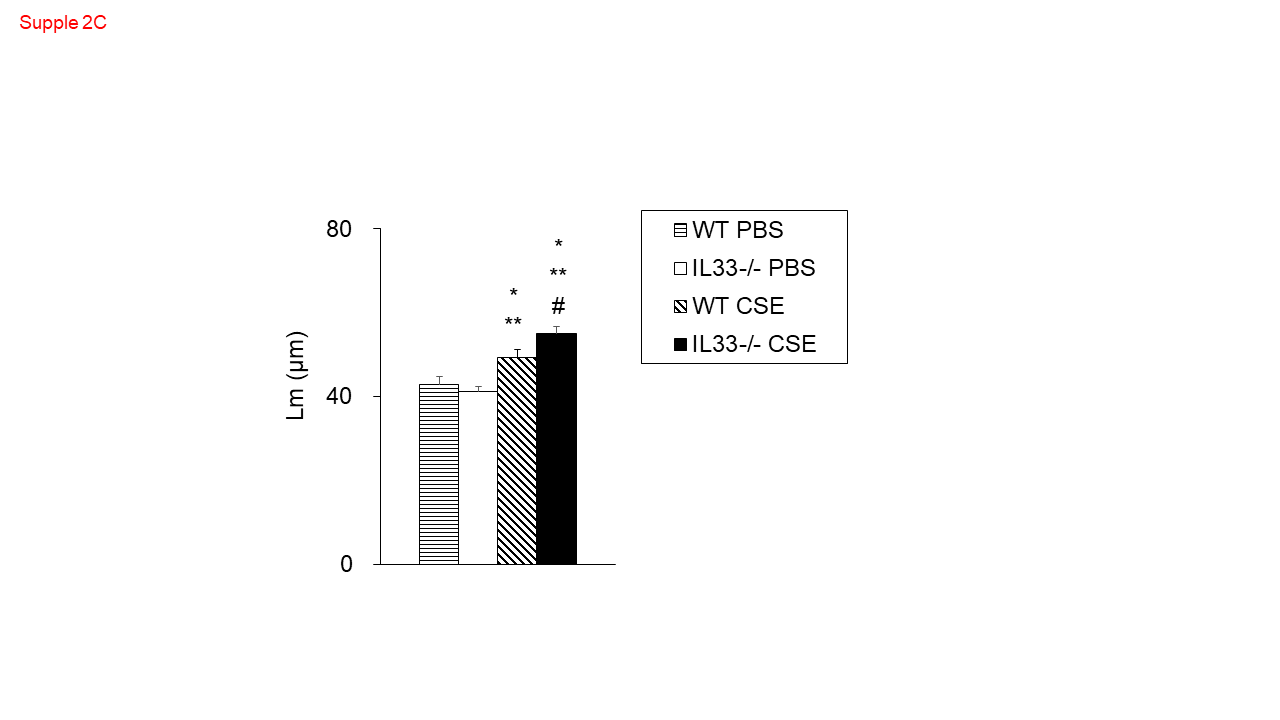

Supplement: Supplementary file 4 — Additional file 4. (C) Lm values. WT PBS: WT mice treated with PBS. IL-33-/- PBS: IL-33-/- mice treated with PBS. WT CSE: WT mice treated with CSE. IL-33-/- CSE: IL-33-/- mice treated with CSE. The results for each group are shown as the mean ± SEM; n = 6–9 in each group. *P < 0.05 compared to WT PBS; **P < 0.05 compared to IL-33−/− PBS; #P < 0.05 compared to WT PPE. [file 12931_2021_1705_MOESM4_ESM.tif]
